# BODY COMPOSITION ACROSS THE LIFESPAN IN AFRICAN CARIBBEAN MEN: FINDINGS FROM THE TOBAGO LONGITUDINAL STUDY OF AGING

**DOI:** 10.1093/geroni/igz038.1802

**Published:** 2019-11-08

**Authors:** Adam J Santanasto, Iva Miljkovic, Ryan K Cvejkus, Allison L Kuipers, Victor Wheeler, Joseph M Zmuda

**Affiliations:** 1 University of Pittsburgh, Pittsburgh, Pennsylvania, United States; 2 Department of Epidemiology, University of Pittsburgh; Pittsburgh, Pennsylvania, United States; 3 Tobago Health Studies Office, Scarborough, Tobago, Trinidad & Tobago, Tobago, Trinidad and Tobago

## Abstract

Body composition changes vary by age and ethnicity and have a major impact on health and physical function. However, little is known about the magnitude, tempo and patterns of these changes in African-ancestry populations, particularly outside the U.S. Thus, we examined age-specific rates-of-change in lean and fat mass in a unique population-based, longitudinal cohort study of 2621 African-ancestry men on the Caribbean island of Tobago (age: 62.0±11.8 years, range: 32-99 years). Body composition was measured with DXA at study entry and after an average of 4 and 9 years. Annualized rates of change and 95% confidence intervals were calculated using all 3 time-points with Generalized Estimating Equations stratified by 5-year baseline age-groups. Lean mass declined at a fairly constant rate in age-strata up through age ≤64 years (-0.72; -0.76, -0.67%/yr), but accelerated to -0.92 %/yr (-1.02, -0.82 %/yr) among those aged 65-69, and to -1.16 %/yr (-1.30, -1.03 %/yr) among those aged 70-74 years – plateauing in those aged 75+. This pattern of age acceleration was observed in arm but not leg lean mass. The age-specific rates of decline in lean mass in this cohort of African Caribbean men appear to be lower than those reported in older African American men. In contrast to lean mas, fat mass increased by 2.93 %/yr (2.72, 3.15 %/yr) and this rate of increase was fairly uniform across the lifespan. Additional research is needed to better define the lifestyle, medical and biological factors contributing to body composition changes across the lifespan in African-ancestry populations.

